# Who is using snus? - Time trends, socioeconomic and geographic characteristics of snus users in the ageing Swedish population

**DOI:** 10.1186/1471-2458-11-929

**Published:** 2011-12-14

**Authors:** Margareta Norberg, Gunnar Malmberg, Nawi Ng, Göran Broström

**Affiliations:** 1 Centre for Population Studies/Ageing and Living Conditions Programme, Umeå University, Umeå SE-901 87, Sweden; 2 Department of Public Health and Clinical Medicine, Epidemiology and Global Health, Umeå University, Umeå SE-901 87, Sweden; 3 Umeå Centre for Global Health Research, Umeå University, Umeå SE-901 87, Sweden; 4 Department of Statistics, Umeå University, Umeå SE-901 87, Sweden; 5 Department of Social and Economic Geography, Umeå University, Umeå SE-901 87, Sweden

## Abstract

**Background:**

The prevalence of smoking in Sweden has decreased in recent decades, and is now among the lowest in the world. During the same period, the use of Swedish moist oral snuff, a smokeless tobacco called snus, has increased. Few studies have evaluated time trends of the socioeconomic and geographic characteristics of snus users in Sweden. This paper contributes to filling that gap.

**Methods:**

This study utilized the Linnaeus Database, which links national registers with comprehensive individual data on socioeconomic status (SES) to health data from a large ongoing health survey, the Västerbotten Intervention Programme (VIP). The VIP targets the entire middle-aged population of Västerbotten county at ages 40, 50 and 60 years with yearly cross-sectional surveys including self-reported data on tobacco habits. Time trends of snus use among 92,563 VIP-participants across different areas of residence and smoking groups were investigated graphically. Logistic regression was performed to estimate the associations between SES and geographical variables and current use versus non-use of snus.

**Results:**

Overall, in parallel to decreasing smoking, the increasing trend of snus use in this middle-aged population continues, particularly in 40-year-olds. In both genders, the highest prevalence of snus use was observed among previous smokers. The prevalence of snus use also increased over time among smokers, and was consistently higher compared to those who had never smoked. Among males - both those who had never smoked and previous smokers - low education (OR 1.21, 95%CI 1.06-1.40 and OR 1.28, 95%CI 1.14-1.43), living alone (OR 1.16, 95%CI 1.07-1.27 and OR 1.13, 95%ci 1.04-1.23), low income and living in rural areas was associated with using snus, while this was not seen among male current smokers. Among women, living alone was associated with using snus irrespective of smoking habits. Among female smokers, the OR for snus use increased with higher education.

**Conclusions:**

A disadvantaged social profile and also higher prevalence in rural areas is observed among male snus users who had never smoked or were previous smokers. Among male smokers there was no association between SES and use of snus. The prevalence of snus use among women is increasing, but is still considerably lower than that of men. The association between snus and SES characteristics is less pronounced among women, although snus is clearly linked to living alone. These patterns should be taken into consideration in tobacco control policies.

## Background

The prevalence of smoking in Sweden has decreased in recent decades, and is now among the lowest in the world [1]. During the same period the use of Swedish moist oral snuff, a smokeless tobacco called snus, has increased. In 2010, 12% of Swedish men and 13% of Swedish women were daily smokers. Smoking was most common in those aged 45-64 years, among whom 18% of men and 17% of women smoked. Overall, snus was used daily by 20% of men and 4% of women, and the corresponding numbers in ages 45-64 years were 22% and 4%, and in ages 30-44 years 23% and 5%, respectively [2]. There is a South to North gradient, with decreasing prevalence of smoking and increasing prevalence of snus use [2]. Overall tobacco use, i.e. including both smoking and snus use, has been stable during the past 20 years [3] but with a decreasing tendency during most recent years [2,4]. The habit of using snus seems to be more stable than smoking, as most men who start using snus also continue [5,6].

Differences between socioeconomic groups with regard to smoking have been shown in numerous studies. Smoking is more prevalent among those with shorter education [7-10], and this may associate to the difference in mortality rates between socioeconomic groups. However, there are few studies that evaluate the characteristics of snus users. Traditionally, in Sweden snus has been used most frequently among blue-collar workers. However, in the 1980s and the first part of the 1990s, the use of snus increased more among men with high education (>12 school years). Despite this, national data in 2010 showed that snus use was still more prevalent among men with only a basic education (<10 school years) and among both men and women with low income [2]. Recently a cross-sectional study from Stockholm, representing 20% of the Swedish population aged 18 years and above, showed that the socioeconomic and life style profiles among snus users are less favourable compared to those of non-users of tobacco, but not to the same extent as among smokers [11]. That study used national register data on education, income, and self-reported data on age and occupation. In both men and women, snus was used most frequently among those 18-24 years old. Among men, those with low or intermediate education, those with low or intermediate income, and those who were skilled workers were at increased risk of being snus users. Among women, snus use was significantly associated with mid-level education, but not with occupational class or income. In both genders, risky alcohol consumption and binge drinking were associated with both snus use and smoking, and the strongest effects were seen among those who were dual users of tobacco. In a study based on the Swedish Twin Registry for the period 1998-2002, male current snus users aged 40+ were more sedentary and had higher alcohol consumption compared to both former snus users and those who had never used snus [12]. However, contrary to results in the study by Engstrom et al. [12], the Swedish male twins were higher educated. In a recent analysis of repeated cross-sectional and longitudinal self-reported data from the Västerbotten Intervention Programme (VIP), we observed that the socioeconomic gap is again widening, with both more smoking and more snus use among those with a basic education compared to those with high education [13].

There is convincing evidence that smoking is the form of tobacco that carries the strongest risk of disease and increased mortality [14,15]. Moreover, smoking is the leading health risk in comparison with other lifestyle habits and with most other consumed products [16]. The health risk of snus use has been intensively debated, and it is proposed that snus use might improve the population's health if sufficient numbers of smokers switch to it [17]. However, despite only a small difference in health-adjusted life expectancy between someone who has never used tobacco compared to a snus user who has never smoked, at the population level a net harm would occur if most snus users would have been tobacco free if they were not using snus [17]. Reviews and meta-analyses conclude that the literature does not provide much evidence of an increased risk among snus users of cardiovascular disease [14] and cancer [15], although there are some indications of an increased risk of fatal CVD [18], oesophageal and pancreatic cancers [19] all-cause mortality [20] and adverse pregnancy outcomes [21-23].

One concern in today's society is the risk of widening socioeconomic gaps in health among ageing cohorts [24], where differences in lifestyle between social groups might result in growing differences in health outcomes in older ages [25,26]. Snus use still is most common in younger generations, among whom the above mentioned chronic diseases are seldom found. However, since tobacco use is one of the key determinants of ill-health, changing patterns of tobacco use, including the use of snus, could be influential to the health status of different social groups when these generations grow older. Snus use could be a pathway from smoking to a less unhealthy form of tobacco use, a way to maintain rather than leave tobacco dependency, or, when going from no tobacco use to snus use, a pathway into a more unhealthy lifestyle. Snus use related to socioeconomic differences may have an impact on the health situation of the middle-aged and the young-old and hence, in the long run, also in older ages. With the ban on snus in the European Union currently under reconsideration, snus is no more a concern only for Sweden, which is the only market for snus in the EU.

More research is needed to better understand the determinants of snus use and its possible health effects. This is also crucial in the effective implementation of tobacco control policies. The aims of this paper are to: (i) evaluate the trends in snus use among the middle-aged population in northern Sweden, which is the region with the highest prevalence of snus use, using data from repeated population-based surveys on tobacco habits; and (ii) assess socioeconomic determinants on snus use patterns over time, utilizing several variables from the national registers on SES. Our hypothesis is that the trends over time of snus use differ, not only between age groups and socioeconomic profiles but also depending on where people live.

## Methods

### Setting and study population

This study is part of the Ageing and Living Conditions Programme (ALC) at Umeå University, in which the socioeconomic differentiation in health in ageing cohorts is a key issue. In order to scrutinize the relationships between health, lifestyle and socioeconomic factors, the Linnaeus Database was developed [27]. On an individual level, this database links records from the administrative registers provided by Statistics Sweden and the National Board of Health and Welfare, including comprehensive data about socioeconomic conditions, hospitalization and causes of death, with the information from the VIP. Västerbotten county in northern Sweden has a population of 260,000 inhabitants, which is concentrated to the coastland, where 44% lives in the city of Umeå, with university and administrative centers, and 28% in the industrial town of Skellefteå. Another 5% reside in Lycksele, a small town which is the commercial center of the inland, and the rest of the population is spread over smaller municipalities and sparsely populated rural areas. The educational level has increased considerably in both men and women during recent decades, in particular among younger adults. The VIP has been underway in the county of Västerbotten in northern Sweden since 1985, when it was launched and developed in one municipality. Beginning in 1990, it was gradually implemented in the rest of the county, and since 1995 all inhabitants in the county are eligible for invitation to a health examination the year they turn 40, 50 or 60 years old. Until 1995, 30-year-olds were also included. The survey is integrated into primary health care routines. Cardiovascular risk markers are measured, and participants answer a comprehensive questionnaire on their health and psychosocial situation as well as life-style habits, including tobacco consumption. Details regarding the design and methodology of VIP have been previously described [28].

This report is based on consecutive yearly cross-sectional VIP surveys for the period 1990-2006, including a total of 100,522 subjects. Since subjects aged 30 years old (n = 7959) were only included in the VIP routine until 1995, this age group was excluded from the analyses. Thus, data from 92,563 health surveys were analysed (men 48.6%). Participation rates were 48-57% during 1990-1995, partly due to intermittent total discontinuance of VIP at some health centres. They then increased and have been at 66-69% since 2005. Previous drop-out analyses showed only minimal social selection bias between participants and non-participants [29].

### Variables

The tobacco habits variables are based on two different questions. The question on smoking is "Do you currently smoke?" with the following seven alternative answers; No I never smoked, Yes I smoke cigarettes, Yes I smoke cigars, Yes I smoke occasionally (not daily), Not now but I used to smoke daily, Not now but I used to smoke occasionally. Smoking was categorized into three categories: never smoked, previous smokers and current smokers. Previous intermittent smokers (used to smoke now and then, not daily) and previous daily smokers were categorized as previous smokers. Smokers who smoked intermittently or daily were grouped together as current smokers. Smokers of cigars or pipe were categorized as smokers. The question on snus was "Have you ever used snus?" with the following six alternative answers; No, Yes I used to but not anymore, Yes I do less than 2 cans/week, Yes I do 2-4 cans/week, Yes I do 5-6 cans/week, Yes I do 7 or more cans/week. Snus use was categorized into use or non-use of snus. Individuals who had quit using snus were categorized as non-users.

To scrutinize the impact of SES on snus use, a number of socioeconomic indicators were selected from the register data provided by Statistics Sweden. Information from the year before participation in VIP was used to ensure that all SES information was collected before the health survey. This ensures the assessment of causation of SES on snus use, which could not be achieved in our earlier cross-sectional investigation [13].

Education was categorized into basic (9 schooling years, compulsory in Sweden), mid-level (10-12 years), and high (13 years or more, i.e. university-level). Yearly income is given in 100 SEK, and this amount is adjusted to the 1990 Swedish retailer price index, thus incomes are comparable over the period irrespective of changes in money value. Employment status is recorded as employed (includes self-employment), or unemployed (defined as a person with recorded unemployment compensation exceeding the recorded income from employment estimated for each year).

Status as pensioner was also recorded. As the VIP only includes those aged up to 60 years, and the normal age for retirement in Sweden is 65 years, in this study population retirement is generally due to disability pension. Family situation was categorized into married/cohabiting versus living alone (includes divorced and widowed). In Sweden cohabiting has a status similar to that of being married, and cohabiting couples who have children together are in the national data registered as cohabiting, while those who do not have children together are registered as singles.

Place of residence was defined as living in one of six geographical areas, one for each of the three cities in the county--the middle-sized city of Umeå with its university and administrative centres, the industrial town of Skellefteå, and the small regional centre of Lycksele; and three different rural areas--a more densely populated coastal area in the east where a large proportion of the population commutes to their work-places in the cities, the less densely populated inland area in the central part of the county, and the most sparsely populated and remote mountain area in the west.

### Statistical analyses

Due to great differences between genders regarding snus use, all analyses were done for men and women separately. Distributions for the categorical variables are presented as percentages. Time trends of snus use were investigated graphically by area of residence and by smoking group. The results were presented in the four time periods of 1990-1994, 1995-1998, 1999-2002 and 2003-2006. Logistic regression was performed to estimate the effects of the explanatory variables with the outcome current snus use versus non-use. There were strong interactions in both men and women between smoking and some of the other covariates; for women education and retirement, and for men education and area of residence. Therefore, the results are presented separately for each smoking group. There were also interactions between periods and education and income. All analyses were done in the statistical computing environment R [30].

### Ethical considerations

Individuals gave informed consent prior to the health screening, and the study was approved by the regional Research Ethics Board at Umeå University (08-131M and 07-142Ö).

## Results

### Characteristics of the study population and trends of snus use

Socioeconomic characteristics of snus and non-snus users, men and women separately, are presented in Table 1. Snus use was considerably more common among men, 26.3%, versus 5.3% among women. In both genders, the use of snus was most frequent in the youngest age groups, among those who lived alone or were unemployed. Among men, the prevalence of snus use was 1.7 times higher among 40-year-olds than among 60-year-olds, and among women it was 5.6 times higher. Among men, those with mid-level education had the highest and those with high education the lowest prevalence of snus use. By contrast, among women, the highest prevalence was observed among those with high education and the lowest among those with low education.

**Table 1:** Socioeconomic characteristics (%) among participants in the Västerbotten Intervention Programme, northern Sweden, 1990-2006.

	Men		Women	
	**Not snus user N = 33 122**	**Snus user N = 11 843**	**Not snus user N = 45 068**	**Snus user N = 2530**

Age group				

40-years	10,017 (67.1)	4907 (32.9)	14,325 (90.5	1499 (9.5)

50-years	11,930 (73.5)	4300 (26.5)	16,264 (95.4)	782 (4.6)

60-years	11,175 (80.9)	2636 (19.1)	14,479 (98.3)	249 (1.7)

Income, 100 SEK/year	2600 (1653, 3270)	2540 (1655, 3121)	1857 (1020, 2402)	1924 (1059, 2441)

Married/cohabiting				

Yes	24,698 (74.4)	8490 (25.6)	33,751 (95.3)	1656 (4.7)

No	8424 (71.5)	3353 (28.5)	11,317 (92.8)	874 (7.2)

Retired				

No	31,071 (73.4)	11,280 (26.6)	41,846 (94.5)	2445 (5.5)

Yes	2051 (78.5)	563 (21.5)	3222 (97.4)	85 (2.6)

Education				

Basic	7923 (75.0)	2641 (25.0)	8105 (97.3)	221 (2.7)

Mid-level	18,327 (71.5)	7306 (28.5)	24,086 (94.2)	1480 (5.8)

High	6857 (78.4)	1888 (21.6)	12,864 (93.9)	829 (6.1)

Unemployed				

No	29,431 (74.2)	10,239 (25.8)	40,444 (94.9)	2166 (5.1)

Yes	3691 (69.7)	1604 (30.3)	4624 (92.7)	364 (7.3)

Smoking *				

Never smoked	17,311 (84.2)	3260 (15.8)	22,100 (98.2)	405 (1.8)

Previous smoker	9579 (62.5)	5750 (37.5)	12,628 (89.0)	1556 (11.0)

Current smoker	6008 (71.5)	2393 (28.5)	9901 (95.1)	506 (4.9)

Place of residence				

Umeå	8826 (75.1)	2930 (24.9)	12,447 (93.6)	848 (6.4)

Skellefteå	5768 (75.4)	1884 (24.6)	8242 (95.3)	406 (4.7)

Lycksele	1856 (72.7)	696 (27.3)	2482 (94.4)	148 (5.6)

Rural east	9305 (73.8)	3303 (26.2)	12,231 (94.9)	654 (5.1)

Rural middle	3973 (71.9)	1551 (28.1)	5171 (95.2)	258 (4.8)

Rural west	3198 (69.2)	1425 (30.8)	4211 (95.2)	211 (4.8)

Periods				

1990-1994	8892 (78.5)	2438 (21.5)	12,485 (98.4)	203 (1.6)

1995-1998	7815 (74.7)	2648 (25.3)	10,870 (96.1)	442 (3.9)

1999-2002	7740 (71.6)	3072 (28.4)	10,316 (93.4)	730 (6.6)

2003-2006	8675 (70.2)	3685 (29.8)	11,397 (90.8)	1155 (9.2)

For income, the median and the first and the third quartiles are given in 100 Swedish crowns (SEK)/year. Numbers and row percents are given for all other variables

* Missing values among non-users and users of snus were 0.7% and 3.7% among men, and 1.0% and 2.5% among women

**Figure 1:**
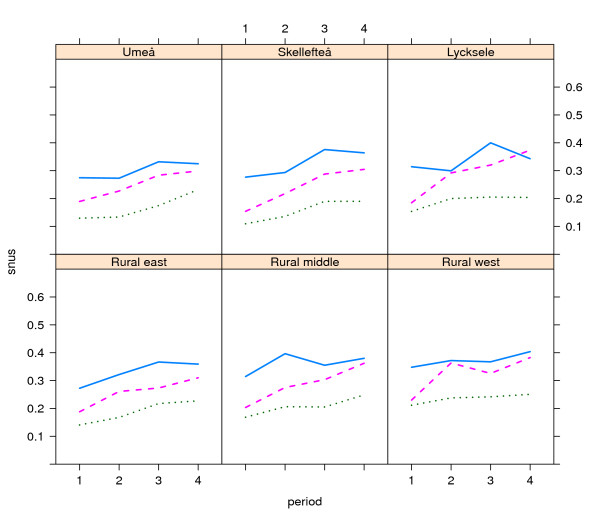
**Trends in snus use by place of residence among men in the Västerbotten Intervention Programme 1990-2006**. The lines show the prevalence of snus use in the three age groups 40 years old (blue unbroken line), 50 years old (red broken line) and 60 years old (green dotted line), by place of residence in the city of Umeå (n = 11,756) and the small towns of Skellefteå (n = 7,652) and Lycksele (n = 2,552), and the east rural area (n = 12,608), middle rural area (n = 4,623) and west rural area (n = 5,524). Lines depict the mean value for the following periods: 1990-1994 (1), 1995-1998 (2), 1999-2002 (3) and 2003-2006 (4).

**Figure 2:**
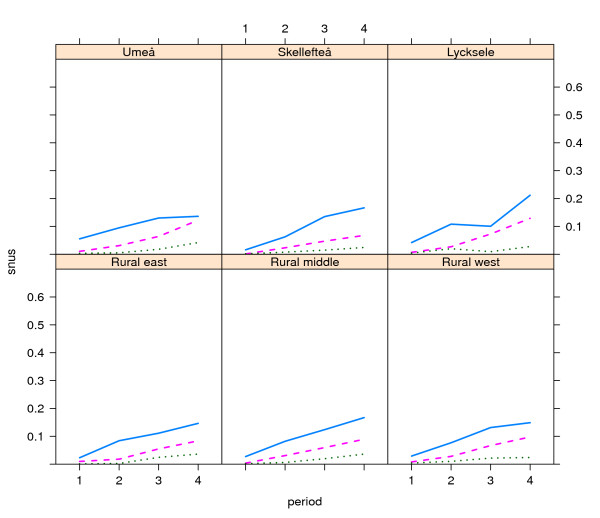
**Trends in snus use by place of residence among women in the Västerbotten Intervention Programme 1990-2006**. The lines show the prevalence of snus use in the three age groups 40 years old (blue unbroken line), 50 years old (red broken line) and 60 years old (green dotted line), by place of residence in the city of Umeå (n = 13,295) and the small towns of Skellefteå (n = 8,648) and Lycksele (n = 2,630), and the east rural area (n = 12,885), middle rural area (n = 4,422) and west rural area (n = 5,429). Lines depict the mean value for the following periods: 1990-1994 (1), 1995-1998 (2), 1999-2002 (3) and 2003-2006 (4).

**Figure 3:**
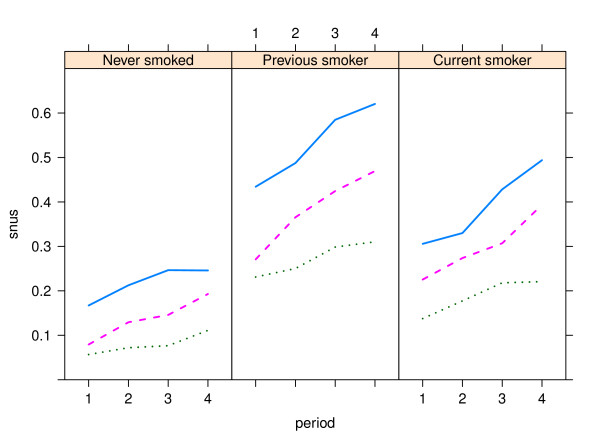
**Trends in snus use by smoking group among men in the Västerbotten Intervention Programme 1990-2006**. The lines show the prevalence of snus use in the three age groups 40 years old (blue unbroken line), 50 years old (red broken line) and 60 years old (green dotted line); and by smoking history - never smoked (n = 20,571), previous smoker (n = 15,329) and current smoker (n = 8,401). Lines depict the mean value for the following periods: 1990-1994 (1), 1995-1998 (2), 1999-2002 (3) and 2003-2006 (4).

**Figure 4:**
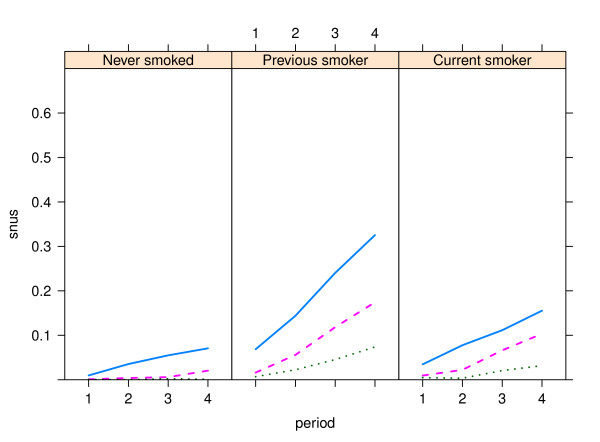
**Trends in snus use by smoking group among women in the Västerbotten Intervention Programme 1990-2006**. The lines show the prevalence of snus use in the three age groups - 40 years old (blue unbroken line), 50 years old (red broken line) and 60 years old (green dotted line), and by smoking history - never smoked (n = 22,505), previous smoker (n = 14,184) and current smoker (n = 10,407). Lines depict the mean value for the following periods: 1990-1994 (1), 1995-1998 (2), 1999-2002 (3) and 2003-2006 (4).

Patterns were similar over time, with increasing snus use in all geographical (Figures 1 and 2) and smoking (Figures 3 and 4) categories, with snus used most frequently in younger ages and considerably more among men than women. Among men, the snus prevalence was generally higher in the rural areas, and during the most recent years (2003-2006) the increase tended to slow in the urbanized areas. Among women the differences were small between geographical regions. The use of snus increased in all female smoking categories, but was most frequent among previous smokers. During the whole period of 1990-2006 and in all ages, snus use was also more prevalent among current smokers compared to those who had never smoked.

An additional analysis among smokers, showed that the proportion who smoked occasionally was stable during the whole study period in both men and women, 19.0-19.9% and 15.1-17.1%, respectively. From 1990-1994 to 2003-2006, the prevalence of snus use increased steadily and slightly among occasional smokers, from 46.8% to 50.0% among men and from 26.6% to 34.4% among women. At the same time dual use among daily smokers increased from 19.6% to 27.7% among men and from 9.8% to15.9% among women.

### Simple logistic regression

The impact of SES on the use versus non-use of snus was first tested for each of the socioeconomic variables separately (data not shown). Among women, income, area of residence, unemployment and retirement did not show statistically significant associations with snus use. This was true among men for the unemployment and retirement variables.

### Multiple logistic regression

In the multiple model, only those variables that were significant were included. Results for men and women are shown in Tables 2 and 3, respectively. Overall, higher ORs with later time period and with younger age were seen in both men and women, regardless their smoking status. Among women, the 95% CI intervals for age were very wide, although significant with *p *< 0.05, due to the low prevalence of snus use in the reference category, i.e. women 60 years of age. Living alone was also associated with an increased risk of snus use in both genders, except for men who were current smokers, and the highest ORs were seen among women living alone. Higher education was associated with snus use among women who were current smokers, but it seems protective against snus use among men who never had smoked or were previous smokers. Further associations between snus use and socioeconomic variables in women were not found. Among men who had never smoked (men who used snus the least, as shown by the intercept 0.38) or were previous smokers (used snus the most, intercept 1.52), there were statistically significant associations with low education, low income and living in rural areas. With regard to geography the ORs were generally higher the longer was the distance from the most urbanized area. The risk of snus use was higher among men who had never smoked in Skellefteå town than among those in Umeå city. In contrast, among male current smokers, the use of snus was not associated with SES characteristics or place of residence, with the exception of a reduced risk of snus use for those living in the town of Skellefteå compared to those living in Umeå city.

**Table 2:** Factors influencing use in different categories of smoking among men. The study population participated in the Västerbotten Intervention programme during the period 1990-2006.

	Never smoked n = 20913 OR 95%CI		Previous smoker n = 15594 OR 95%CI		Current smoker n = 8749 OR 95%CI	
Intercept	0.38	0.33-0.44	1.52	1.33-1.74	0.63	0.53-0.75

						

Age group						

60	1		1		1	

50	**1.99**	1.76-2.25	**1.78**	1.64-1.93	**1.86**	1.63-2.12

40	**3.39**	3.02-3.81	**3.29**	3.00-3.61	**2.82**	2.45-3.24

Education						

High	1		1		1	

Mid-level	**1.25**	1.12-1.38	**1.20**	1.09-1.32	1.06	0.91-1.24

Basic	**1.21**	1.06-1.40	**1.28**	1.14-1.43	1.10	0.92-1.31

Married/Cohabiting						

Yes	1		1		1	

No	**1.16**	1.07-1.27	**1.13**	1.04-1.23	0.98	0.88-1.09

						

Place for residence						

Umeå	1		1		1	

Skellefteå	**1.19**	1.05-1.34	0.98	0.88-1.10	**0.77**	0.65-0.90

Lycksele	1.16	0.97-1.39	1.13	0.96-1.32	1.20	0.96-1.50

Rural east	**1.13**	1.01-1.26	1.03	0.93-1.12	0.97	0.84-1.10

Rural middle	**1.43**	1.25-1.64	**1.13**	1.01-1.28	1.06	0.83-1.17

Rural west	**1.67**	1.45-1,92	**1.15**	1.02-1.31	1.13	0.95-1.34

						

Income (100SEK)	**0.91**	0.89-0.94	**0.96**	0.94-0.99	1.03	0.99-1.07

						

Year	**1.06**	1.05-1.07	**1.06**	1.05-1.07	**1.06**	1.05-1.07

Multivariate logistic regression analyses* with snus use versus non-use as the outcome in the three smoking categories; Never smoked, Previous smoker and Current smoker

* Only variables with significant odds ratios (ORs) with 95% confidence intervals (95% CI) are presented Significant ORs are marked in bold. The following variables were not included in the multivariate analyses due to non-significance in the univariate analyses: Unemployment, retirement

**Table 3:** Factors influencing use in different categories of smoking among women. The study population participated in the Västerbotten Intervention programme during the period 1990-2006.

	Never smoked n = 23125 OR 95%CI		Previous smoker n = 14568 OR 95%CI		Current smoker n = 10860 OR 95%CI	
Intercept	0.00	0.00-0.00	0.03	0.02-0.03	0.02	0.01-0.03

						

Age group						

60	1		1		1	

50	**8.50**	4.13-20.6	**2.71**	2.28-3.24	**3.13**	2.26-4.44

40	**48.4**	24.6-114	**7.09**	5.99-8.44	**6.79**	4.92-9.60

Education						

High	1		1		1	

Mid-level	1.11	0.90-1.37	0.99	0.88-1.12	**0.61**	0.49-0.75

Basic	1.04	0.60-1.68	1.01	0.82-1.23	**0.36**	0.25-0.51

Married/Cohabiting						

Yes	1		1		1	

No	**1.60**	1.28-2.00	**1.57**	1.40-1.77	**1.34**	1.11-1.62

						

Year	**1.16**	1.13-1.19	**1.17**	1.15-1.18	**1.16**	1.14-1.19

Multivariate logistic regression analyses* with snus use versus non-use as the outcome in the three smoking categories; Never smoked, Previous smoker and Current smoker

* Only variables with significant odds ratios (ORs) and 95% confidence intervals (95% CI) are presented. Significant ORs are marked in bold. The following variables were not included in the multivariate analyses due to non-significance in the univariate analyses: Income, area of residence, unemployment, retirement

## Discussion

This study from northern Sweden draws a picture of snus users as belonging to the part of the population with a less advantaged socioeconomic situation. This is most evident among men who are previous smokers as well as men who have never smoked, among whom snus users are characterized by lower income, shorter education and living alone. These factors have little impact on the use of snus versus non-use among smoking men - who, regardless of snus use, constitute a group with lower SES [8]. Among women, socioeconomic characteristics seem to have little influence on the use of snus, although we found an increased risk among women who live alone, and, for smoking women also among those with high educational level. These findings are consistent with previous cross-sectional national studies [2], as well as with studies from both Sweden's most populated area, Stockholm [11], and from southern Sweden [31,32]. We contribute with results from a considerably larger study population in the North of Sweden and by showing trends in snus use over time. Snus use increased in all groups during the study period 1990-2006, but tended to plateau during the most recent years among men who live in urbanized areas. The urban-rural gradient among men, with more prevalent snus use in the least urbanized areas, persisted after income, education and other SES characteristics were controlled for. This is in line with previous studies [32] and findings from national surveys [2].

In Västerbotten county there are active tobacco prevention programs organized on almost all nine-year compulsory schools. In most primary care centers as well as in dental care clinics, in both urban and rural districts, there is access to smoking cessation services, and there are tobacco cessation clinics in the three hospitals (Umeå, Skellefteå and Lycksele) (personal communication with the coordinator of Tobacco prevention, Västerbotten county council). Therefore differences in tobacco habits should not to a large extent be due to differences in accessibility of support or resources for tobacco cessation.

In Sweden, the use of snus has long been considered a typical rural habit and, particularly among men. The use of snus is possible during work in manual occupations, which is more common in rural areas, simply because snus is kept under the upper lip for longer periods while cigarettes are hand-held. Thus, although the prevalence of snus use is higher in the north compared to the south of Sweden [2], the socioeconomic pattern among snus users is consistent over the whole country. The associations between education and snus may be due to the well educated being more sensitive to information about health risks, and also to tobacco use in Sweden being related to class-specific lifestyle behaviours. Taken together, these factors might explain why snus use in Sweden is more prevalent among men and shows a clear social gradient and also is more common in rural areas among men, while snus use seems to be without an evident social gradient among women. Because tobacco is expensive, one might assume that people with low incomes would be less prone to use it. However, snus - which is considered cheaper than smoking - might be a substitute for smoking in contexts where it is socially accepted. We found no association between unemployment or disability pension and snus use. However, the economic consequences of these states should at least to some extent be captured in the income variable.

Marriage is shown to be associated with healthier living [33] and also to be protective against smoking, with regard to both continuous use and changes in smoking [34,35]. Our finding of an increased risk of snus use among those who live as singles supports the idea that snus is another aspect of less healthy behaviours in this group.

The link between SES and health [26] and health behaviours [36] has long been well established. The pathways between SES and smoking are suggested to be due to position (education, income, occupation) and psychosocial stressful environments, which in turn have an impact on lifestyle habits [37]. Our results and previous studies [2,11,31,32] show that this also holds true for snus with regard to socioeconomic position. To explore the mechanisms behind the link between snus use and SES is, however, not within the scoop of this study, and would also need other methodologies, preferably qualitative methods, to clarify. In this study we did not include additional lifestyle variables. However, in a previous longitudinal study based on the same study population, we found that high consumption of snus was independently associated with obesity and hypertriglyceridemia, and with metabolic syndrome [38]. Several other studies also document that snus is associated with obesity [31,39,40]. Thus, current knowledge shows that snus is an indicator of unfavourable socioeconomic situation and is related to other unhealthy lifestyles and worsened health outcomes. Based on this, more public health attention to the use of snus is warranted, e.g. clear information, in particular to the younger generations, about possible harmful health effects from snus. This should also be taken into consideration in tobacco control policies.

We also found that the use of snus is not only most prevalent among previous smokers, but also considerably more prevalent among current smokers as compared to those who have never smoked. The additional analysis among smokers also showed that in both genders, those who smoke occasionally constitute a stable group of around one fifth of the smokers, and around 50% of male and one third of female occasional smokers are dual users. The majority of smokers are daily smokers and among them the increase of dual use was more pronounced compared to occasional smokers and of the same magnitude as among previous smokers. Thus, dual use among intermittent smokers, can only to a small degree explain the increase of dual use among smokers (intermittent+daily smokers). This might be surprising, as snus is largely expected to replace smoking and thus lead to a reduction in smoking and reduced harm from smoking [17,41]. What we see is that dual use is on the rise among smokers, similar to other groups, and is now rather common, particularly among 40-50-year-old male smokers, among whom 40-50% also used snus during the most recent years. Although snus might increase smokers' chances of success in quitting among those who use it as a quitting aid [42], several studies also show that most smokers who quit, do so without any snus use [4,42]. Based on our data we cannot evaluate whether dual use among intermittent smokers is associated with smoking fewer cigarettes or only smoking more seldom. However, dual use is associated with strong nicotine addiction [43,44]. Therefore, the role of snus in intermittent smoking and vice versa, and particularly the role of snus in continuing smoking intermittently or daily rather than quitting, as well as the role of dual use in relation to other lifestyle habits, should be the object of further longitudinal studies.

During 2003-2006 in this middle-aged population, snus was used by 20% of males who had never smoked and were aged 50 years, and by 10% of 60-year-olds in this same group. This raises concern with respect to health effects in the ageing population, as it has been shown that among the older group, (aged 45+), the fraction of smoking initiation attributable to snus use is higher than that of smoking cessation [45], which suggests that frequent snus use among "older middle aged" generations might be a gateway into smoking more frequently, and thus leading to a net harm effect rather than harm reduction. Future studies therefore should evaluate possible health effects of snus use among the elderly.

Our results are discrepant to previous studies from this region that document a considerably lower prevalence of dual use [3,6], which might partly be explained by differing definitions of current smoking. We included intermittent smokers in this category, because even a low dose of smoking is a health risk [46], while other studies categorize intermittent smokers as non-smokers [3,6,41]. This report therefore sheds light upon combined intermittent smoking and snus use as a form of dual use.

This study has the strength of combining high quality national statistics on SES with a very large, longstanding population-based health survey, performed in the stable structure of health care using standardized questionnaires regarding tobacco habits. In addition, the Linnaeus database allows us to link SES data from one year preceeding the year of the health survey, which reassures the temporal association between SES and the use of snus, which a cross-sectional study cannot address. As tobacco habits are complex and people change between categories, large and longitudinal study samples are needed to be able to evaluate snus use, not least among women. We are not aware of any other study population that includes 2,500 women and 11,800 men who are using snus. The participation rate varied during the study period, and low participation rates, in particular during the early 1990s, might have resulted in underreporting of tobacco use and thus underestimation of the prevalence of smoking and snus use. However, the relatively low rates in the early 1990s were largely due to temporary discontinuation of VIP at some health care centres, rather than to non-response. Therefore, the non-participant group during these years also contained those who (without any selection) were eligible but not invited. Non-participation among tobacco users, at least in postal questionnaires, might lead to an overestimation of SES differences and an underestimation of smoking prevalence, and this should also be considered in this study [47]. However, in this study participants answered the questionnaire at the health care centre on the same occasion as their examination and dialogue with a trained nurse regarding their own health and lifestyle, and correct answers to the questions is therefore in the participant's own interest. We therefore believe that non-response among smokers is lower in this study than occurs in postal interviewing. A previous study of participation in a 10-year follow-up in the VIP found that the base line prevalence of smoking among non-participants versus participants in the follow-up was 30.8% versus 24.8%. The prevalence of snus use was 28.7% versus 25.6%, a difference of around 3% [48]. This might reflect that snus is generally well accepted in Sweden and the general opinion is that snus does not have a big impact on a person's health. We conclude that snus use should only be associated to a small extent with non-participation, and therefore our results regarding snus use should be reliable. The steady increase of snus use in Sweden is most marked among young adults below 40 years of age, and a limitation of this study is that it only includes middle-aged men and women aged 40-60 years.

## Conclusions

The increasing trend of snus use continues, but has recently slowed among men living in urbanized areas. Snus use is clearly more prevalent among current smokers compared to those who have never smoked. Although the prevalence of smoking is low in this middle-aged population, among smokers dual smoking and snus use is increasing in all age-groups. Among male non-smokers, both previous smokers and those who have never smoked, a disadvantaged social profile with lower income, shorter education, living alone and living in rural areas is associated with the use of snus. In the group of male smokers, SES was similar among users and non-users of snus. Among women, who still use snus to a considerably lower proportion than men, we found a clear link between snus use and living alone, while there were limited differences with regard to other SES characteristics between users and non-users of snus. These patterns of socioeconomic situation along with previous findings of associations between snus and unhealthy lifestyle habits, should be a concern for public health actions and also taken into consideration with regard to tobacco control policies.

## Competing interests

The authors declare that they have no competing interests.

## Authors' contributions

All authors contributed to the conception and study design. MN was involved in the data analyses and interpretation, and drafted the manuscript. GB managed the data and statistical analyses, provided statistical expertise and contributed to the interpretation of the data and drafting the manuscript. NN was involved in the data interpretation and provided critical review of the manuscript. GM contributed to the conceptual description of socioeconomic variables, interpretation of data, and drafting and revision of the manuscript. All authors have read and approved the final manuscript.

## Pre-publication history

The pre-publication history for this paper can be accessed here:

http://www.biomedcentral.com/1471-2458/11/929/prepub
